# DESIGN, EVALUATION, AND IMPLEMENTATION OF THE PRISM SOFTWARE SYSTEM: A SOFTWARE SYSTEM DESIGNED FOR OLDER ADULTS

**DOI:** 10.1093/geroni/igae098.0038

**Published:** 2024-12-31

**Authors:** Sara Czaja, Hans-Werner Wahl, Neil Charness

**Affiliations:** Chair; Co-Chair; Discussant

## Abstract

The PRISM system is an integrated software system designed, using a user-centered design approach, to foster social connectivity, cognitive engagement, and resource access among aging adults. Versions of PRISM have been evaluated in diverse contexts and geographic regions, and with diverse populations. This session will discuss how PRISM has been adapted, and present data on the impact of the use of PRISM across diverse contexts, on outcomes related to technology acceptance and proficiency, social isolation and loneliness, and quality of life. Walter R. Boot will discuss the evolution of PRISM and highlight how the findings from the PRISM 1.0 trial led to the development of PRISM 2.0 and PRISM CI for aging adults with a cognitive impairment. He will also discuss how PRISM was adapted for implementation in Germany. Sara J. Czaja will present the primary outcomes from the PRISM 2.0 trial, which evaluated a refined version of PRISM with older adults in diverse settings (rural locations, senior housing, assisted living facilities). She will focus on outcomes related to loneliness, social isolation, and quality of life. Wendy A. Rogers will focus on the acceptability and usability of PRISM. She will present data from the PRISM 1.0 and PRISM 2.0 trials and highlight findings regarding acceptability according to context of implementation. Nicole Memmer will address long-term usage of PRISM Germany. She will discuss differential patterns and preferences of use in relation to gender, technology generation, and technology skills and attitudes. N. Charness will serve as a discussant for the session.

